# Updated Evaluation of the Diagnostic Performance of Double Contrast-Enhanced Ultrasonography in the Preoperative T Staging of Gastric Cancer: A Meta-Analysis and Systematic Review

**DOI:** 10.3389/fonc.2022.844390

**Published:** 2022-03-09

**Authors:** Xin Zhang, Jun Yao, Yu Zhang, Xin Huang, Weijun Wang, Hejing Huang

**Affiliations:** ^1^ Department of Gastrointestinal Surgery, Second Affiliated Hospital of Naval Medical University, Shanghai, China; ^2^ Department of Ultrasound, Second Affiliated Hospital of Naval Medical University, Shanghai, China

**Keywords:** double contrast-enhanced ultrasonography, gastric cancer, preoperative evaluation, tumor staging, diagnostic performance

## Abstract

**Objective:**

This study aimed to systematically evaluate the diagnostic performance of double contrast-enhanced ultrasonography (DCEUS) in the preoperative T staging of gastric cancer (GC).

**Methods:**

Literature searches for eligible studies were performed using MEDLINE, EMBASE, and Cochrane Library. The pooled sensitivity, specificity, positive likelihood ratio, negative likelihood ratio, diagnostic odds ratio, and area under the summary receiver operating characteristic curve of DCEUS in the diagnosis of each T stage tumor were calculated. Meta-analyses were performed to obtain the pooled effects of risk ratio (RR) with 95% confidence interval (CI) in the comparison of DCEUS with CT/endoscopic ultrasound (EUS).

**Results:**

A total of 8 studies including 1,232 patients were identified for inclusion in this meta-analysis. The pooled sensitivity and specificity were 0.78 (95% CI = 0.64–0.88) and 0.98 (95% CI = 0.96–0.99) for T1, 0.81 (95% CI = 0.76–0.86) and 0.96 (95% CI = 0.91–0.98) for T2, 0.88 (95% CI = 0.84–0.91) and 0.85 (95% CI = 0.79–0.90) for T3, and 0.81 (95% CI = 0.69–0.89) and 0.96 (95% CI = 0.93–0.97) for T4. Moreover, DCEUS demonstrated significant superiority to CT in diagnosing T1 (RR = 1.57, 95% CI = 1.20–2.05, *p* = 0.001) and T2 (RR = 1.41, 95% CI = 1.16–1.71, *p* = 0.001) and to EUS in diagnosing T3 (RR = 1.24, 95% CI = 1.08–1.42, *p* = 0.003) and T4 (RR = 1.40, 95% CI = 1.09–1.79, *p* = 0.008). However, it showed a lower diagnostic accuracy than EUS in T1 tumors (RR = 0.77, 95% CI = 0.62–0.94, *p* = 0.013).

**Conclusions:**

DCEUS is a feasible complementary diagnostic tool for clinical T staging of GC. However, it is still far from a definitive conclusion for DCEUS to be proposed for use in routine clinical practice.

## Introduction

Gastric cancer (GC) represents one of the most common causes of cancer death worldwide (1). As a shift toward a more individualized, stage-dependent treatment of GC has been advocated, accurate preoperative staging is essential for appropriate treatment (2). In particular, the depth of primary tumor invasion, namely, T stage, is both an important indicator for predicting prognosis and a major factor for the determination of an optimal therapeutic strategy (3, 4). Therefore, it is important to explore reliable and effective techniques for preoperative T staging of GC.

Many imaging modalities, such as computed tomography (CT), magnetic resonance imaging (MRI), and endoscopic ultrasound (EUS), have been utilized clinically for evaluating the T stage of GC (5, 6). Particularly, multi-detector row CT (MDCT) with multi-planar reformatted views is believed to be a powerful tool, but its sensitivity in T staging for early GC is low (7). Moreover, it carries a burden on ionizing radiation, which may be an obvious disadvantage. MRI seems to have better performance for high resolution, but the relatively expensive fees and longer scanning time also limit its extensive application in the staging of GC (6). EUS is regularly applied to stage GC due to its high sensitivity (8, 9). However, overstaging of T2 lesions appears to be a frequent problem (10), and EUS could not avoid bringing about some discomfort.

Double contrast-enhanced ultrasonography (DCEUS) refers to the combination of oral contrast agent and intravenous contrast agent for ultrasound examination (11). It has been explored as an innovative modality to screen diseases of the gastrointestinal tract (12). SonoVue is an intravenous contrast agent of sulfur hexafluoride microbubbles, and DCEUS provides a feasible way to make an accurate T staging by using ultrasonic oral contrast agent alongside SonoVue in patients with GC (13). Although there have been some studies that estimated the diagnostic performance of DCEUS in the preoperative T staging of GC (14, 15), only a small number of patients were included in each study. In addition, the only one previously published meta-analysis on the diagnostic accuracy of DCEUS in the T staging of GC is scarce and not robust to reach definitive conclusions (16). Therefore, we aim to provide an updated and revised version of the meta-analysis to determine the diagnostic performance of DCEUS for T staging in patients with GC.

## Methods

### Literature Search

Databases including MEDLINE, EMBASE, and the Cochrane Library were searched up to December 23, 2021 to identify pertinent citations. The following search strategies were employed: (double contrast-enhanced ultrasonography OR double contrast-enhanced ultrasound) AND (stomach OR gastric) AND (cancer OR carcinoma OR neoplasia OR tumor OR adenocarcinoma). For unpublished data, trial registries including clinical trial.gov, the national research register, and current controlled trials were searched. Additionally, a manual search was performed by checking the reference lists in recent important publications. This review involved only the secondary use of anonymous information or anonymous biological materials and thus was exempted from research ethics board review.

### Study Selection

Both prospective and retrospective studies examining the diagnostic performance of DCEUS for the preoperative T staging of GC were included, namely, (1) gastric carcinoma as proven by endoscopic biopsy; (2) without history of chemotherapy, radiotherapy, targeted therapy, immunotherapy, or other cancer-related treatment; (3) the patients were examined by DCEUS not more than one week before the surgical resections; and (4) no age or gender restrictions. Studies were included regardless of the publication date, publication status, and language. The exclusion criteria were as follows: (1) unresectable lesions with metastasis detected on preoperative evaluation, (2) patients medically unfit for surgery, (3) letters to the editor, case reports, editorials, and review articles, (4) studies that did not provide sufficient data to determine at least one of the preoperative staging performance measures (sensitivity, specificity, or accuracy), and (5) studies that did not use the TNM classification system.

The title and the abstract of each article were screened and assessed independently against the predetermined inclusion criteria by two reviewers (XZ and HH). A third party was involved in the discussion and decision-making. A reason must be given for excluding any article.

### Quality Assessment

Two authors (XZ and JY) independently evaluated the overall quality of the included studies by using the Quality Assessment of Studies of Diagnostic Accuracy-2 (17). This method has four domains, namely, patient selection, index test, reference standard, and flow and timing. Each domain was assessed considering the risk of bias, and the first three domains were assessed to confirm the applicability. Each domain contains three judgments, namely, “low”, “high”, and “unclear”. Discrepancies between the two authors were resolved by a discussion. The final results were reviewed by the other authors. The quality assessment of the included study was performed using RevMan 5.3 (Cochrane Collaboration).

### Statistical Analysis

A bivariate model was used to pool the sensitivity, specificity, positive likelihood ratio (PLR), negative likelihood ratio (NLR), and diagnostic odds ratio (DOR) of the included studies (18). A summary receiver operating characteristic (SROC) curve was generated, and the area under the curve (AUC) was calculated to determine the overall diagnostic accuracy of DCEUS (19). Deeks’ test was applied to assess the potential publication bias (20). Fagan graph was plotted to estimate the posttest probability. Heterogeneity across all eligible studies was estimated by using *Q*-test and *I* ^2^ statistics (21). Statistical analyses were performed using STATA 16.0 (StataCorp, College Station, TX, USA) and Meta-DiSc (version 1.4).

## Results

### Description of the Studies

**Figure 1:**
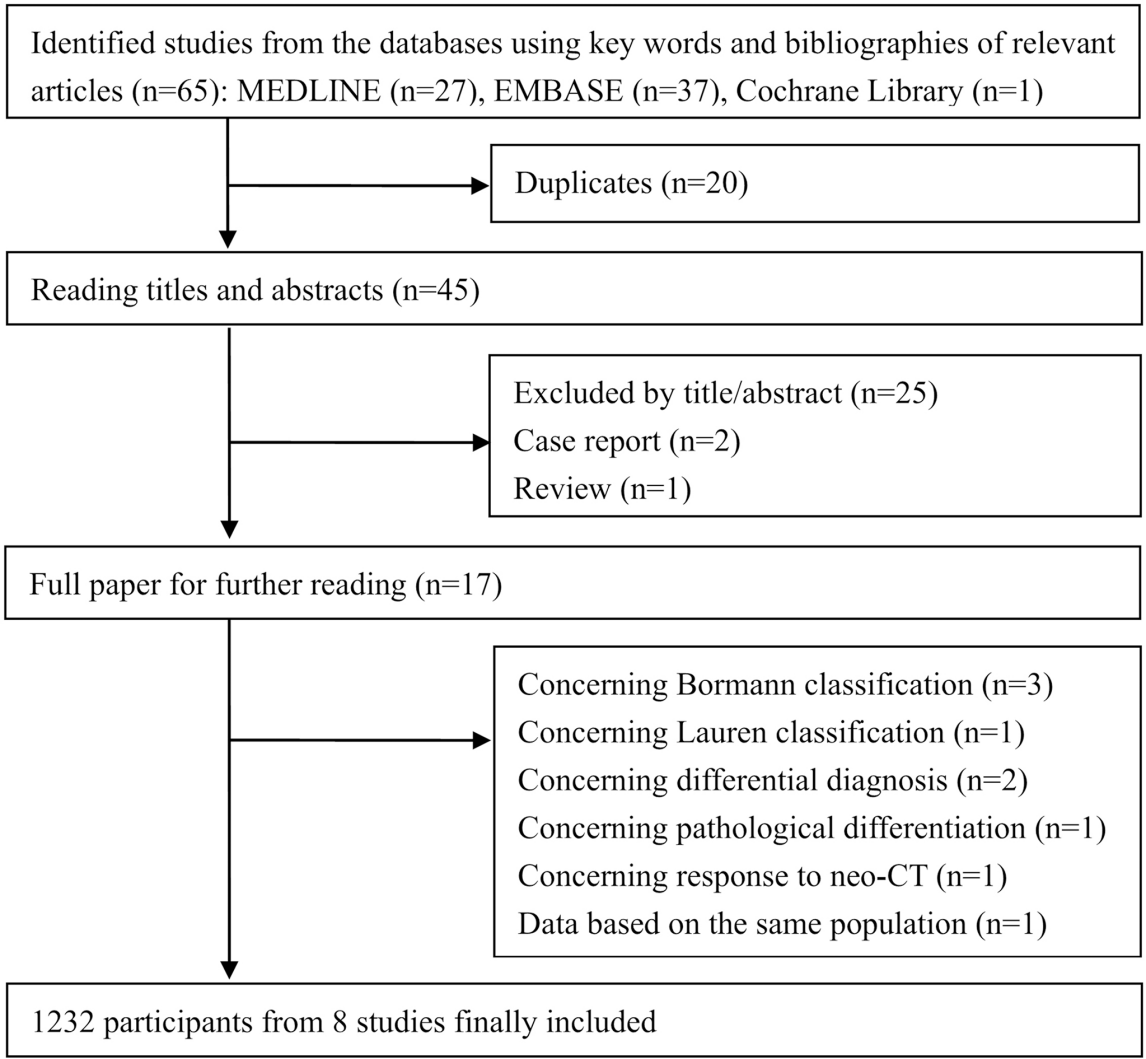
Flow of studies through the review process. neo-CT, neoadjuvant chemotherapy.

**Figure 2:**
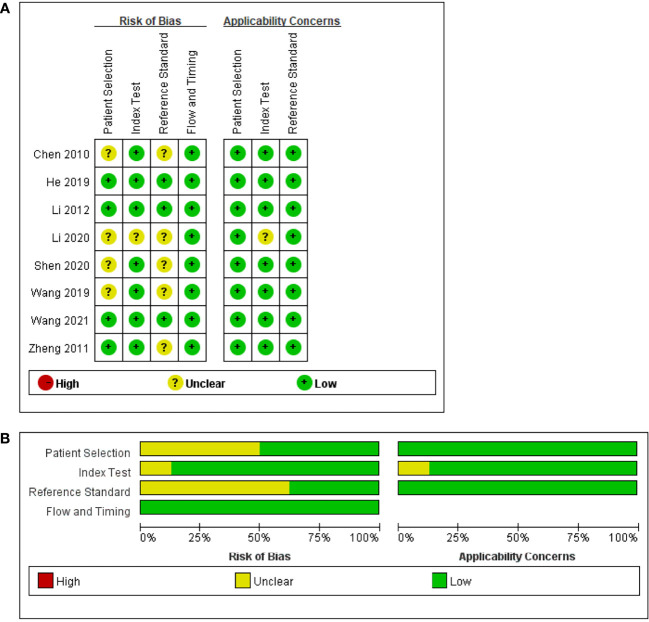
Risk of bias and applicability concerns. **(A)** Summary: review authors’ judgments about each domain for each included study. **(B)** Review authors’ judgments about each domain presented as percentages across the included studies.

A total of 65 citations from database searching were initially identified, of which 20 duplicates were excluded. Seventeen papers were retrieved for full-text review after excluding 25 articles on the basis of the titles and the abstracts, two case reports, and one review article. Nine studies concerning the application of DCEUS irrelevant of preoperative T staging of GC were also further excluded. A total of 8 studies (13–15, 22–26) including 1,232 patients were finally included in this meta-analysis. Of these, seven studies were retrospective, and 1 study was prospective. The publication year ranged from 2010 to 2021. The publication language was English in 6 studies and Chinese in 2 studies. The specific flow chart in identifying eligible studies is shown in **Figure 1**. The main characteristics of the included studies are shown in **Table 1**. The overall quality of the included studies was moderate to high, and the results of the methodological quality assessment (bias risk and applicability) are shown in **Figure 2**.

**Table 1:** Characteristics of the studies included.

Author	Region	Gold standard	PL*	Study type	Number	Gender (F/M)	Age, years	TNM edition	Histopathological composition	UOCA volume, ml	Equipment
Wang (22)	China	Pathology	E	R	206	95/111	59.7 ± 11.3	AJCC, 8th	NS	NS	Acuson Sequoia-512
Shen (23)	China	Pathology	C	R	59	14/45	51.4 ± 10.7	NS	NS	500–800	NS
Li (14)	China	Pathology	E	R	100	42/58	62.3 ± 2.6	AJCC, 8th	Well, 10; moderately, 21; poorly, 66	500–800	NS
Wang (15)	China	Pathology	E	R	158	52/106	59.5 ± 10.6	NS	Well, 22; moderately, 33; poorly, 65; signet ring, 16; mucinous, 8; squamous carcinoma, 1	500	Acuson Sequoia-512
He (13)	China	Pathology	E	R	54	18/36	61.0 ± 9.7	AJCC, 7th	NS	500–800	Philips iU22
Li (24)	China	Pathology	E	P	350	105/245	63.6 ± 11.8	AJCC, 6th	NS	500	Acuson Sequoia-512
Zheng (25)	China	Pathology	E	R	162	35/127	58.3 ± 11.3	NS	Well, 34; moderately, 31; poorly, 68; signet ring, 29	500	Acuson Sequoia-512
Chen (26)	China	Pathology	C	R	143	54/89	56.0 ± 11.4	NS	NS	600	Acuson Sequoia-512

*PL, publication language; C, Chinese; E, English; P, prospective; R, retrospective; UOCA, ultrasonic oral contrast agent; NS, not specified.

### Descriptive Diagnostic Performance of DCEUS

The pooled sensitivity, specificity, PLR, NLR, and DOR of DCEUS in diagnosing each T stage tumor were calculated. The pooled sensitivity and specificity were 0.78 (95% CI = 0.64–0.88) and 0.98 (95% CI = 0.96–0.99) for T1 tumors (**Supplementary Figure S1**), 0.81 (95% CI = 0.76–0.86) and 0.96 (95% CI = 0.91–0.98) for T2 tumors (**Supplementary Figure S2**), 0.88 (95% CI = 0.84–0.91) and 0.85 (95% CI = 0.79–0.90) for T3 tumors (**Supplementary Figure S3**), and 0.81 (95% CI = 0.69–0.89) and 0.96 (95% CI = 0.93–0.97) for T4 tumors (**Supplementary Figure S4**). For each outcome, the pooled PLR, NLR, and DOR are listed in **Table 2**.

**Table 2:** Descriptive diagnostic performance of DCEUS.

	T1	T2	T3	T4
Sen	0.78 (0.64–0.88)	0.81 (0.76–0.86)	0.88 (0.84–0.91)	0.81 (0.69–0.89)
Spe	0.98 (0.96–0.99)	0.96 (0.91–0.98)	0.85 (0.79–0.90)	0.96 (0.93–0.97)
PLR	46.3 (17.5–123.0)	21.1 (8.6–51.4)	5.9 (4.2–8.4)	19.1 (12.1–30.0)
NLR	0.22 (0.12–0.39)	0.19 (0.15–0.25)	0.14 (0.11–0.18)	0.20 (0.12–0.34)
DOR	210 (54–813)	109 (39–309)	41 (26–67)	95 (43–208)

Data are shown as mean (95% confidence limits).

DCEUS, double contrast-enhanced ultrasonography; Sen, sensitivity; Spe, specificity; PLR, positive likelihood ratio; NLR, negative likelihood ratio; DOR, diagnostic odds ratio.

Between-study heterogeneity was high in the pooled sensitivity in T1 (*I* ^2^ = 65.7%, *p* = 0.01) and T4 (*I* ^2^ = 71.1%, *p* < 0.001) and in the pooled specificity in T2 (*I* ^2^ = 85.2%, *p* < 0.001) and T3 (*I* ^2^ = 66.5%, *p* < 0.001). Interestingly, heterogeneity was obviously reduced when the study by He et al. (13) was excluded from the pooled analyses.

The sensitivity analyses were implemented by omitting the included studies one by one. With the sequential removal of each individual study, the overall results were essentially unchanged, indicating the robustness of these findings (**Supplementary Figure S5**).

### Threshold Effect and SROC of DCEUS

**Figure 3:**
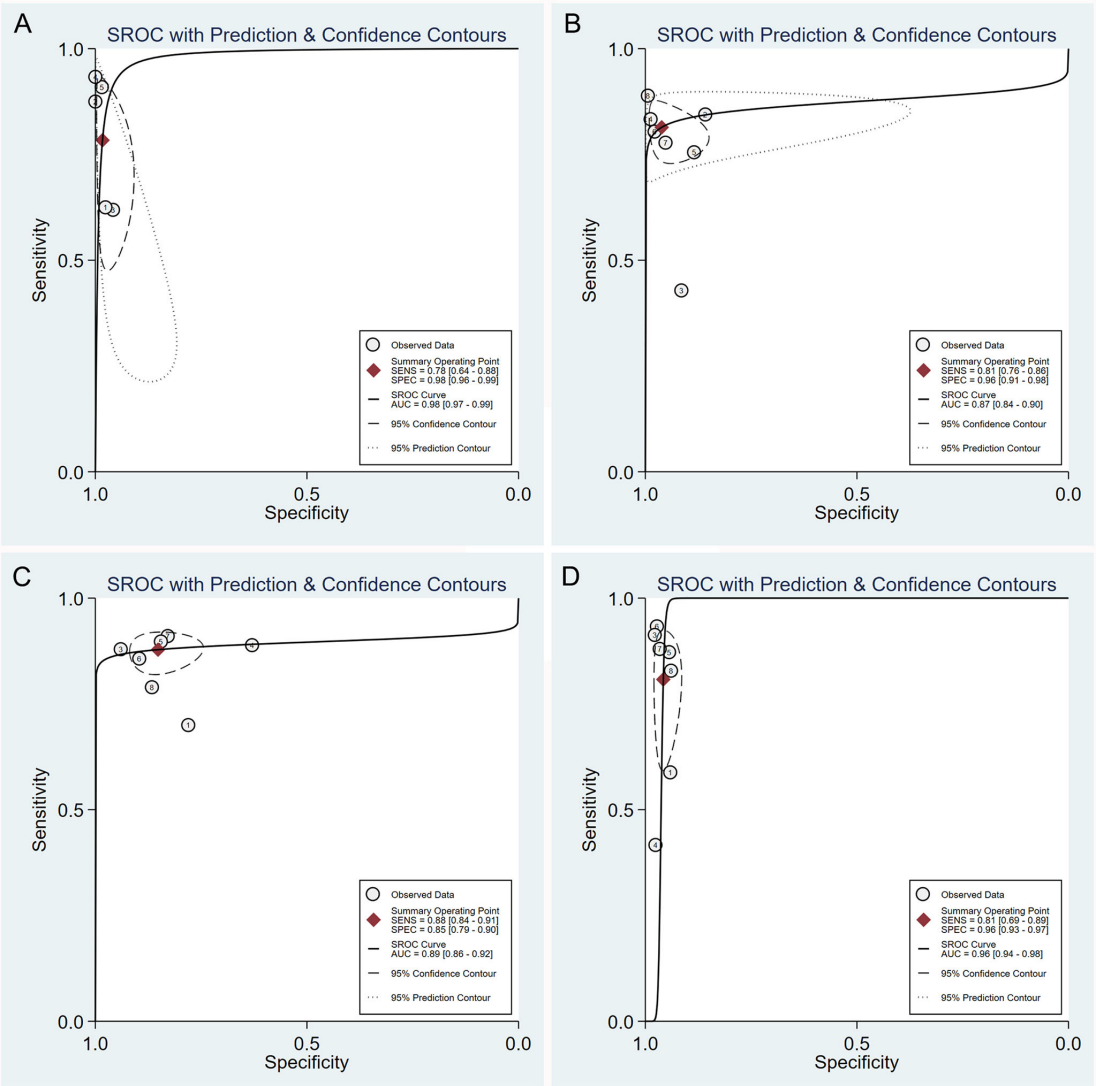
Summary receiver operating characteristic plot of studies assessing the accuracy of double contrast-enhanced ultrasonography in diagnosing T1 **(A)**, T2 **(B)**, T3 **(C)**, and T4 **(D)** gastric tumors. Each study sensitivity/specificity value is represented by an empty circle. The summary point for sensitivity/specificity is represented by a black-filled circle. Dotted closed line, 95% confidence interval of the summary point; dashed closed line, 95% prediction region.

No typical “shoulder arm” was observed in the SROC curve plane graphs for the test of T1, T2, T3, or T4 (**Figure 3**). The correlation coefficients of the sensitivity logarithm were -0.600, -0.429, 0.143, and -0.357, and the corresponding *p*-values were 0.285, 0.337, 0.760, and 0.432 for the test of T1, T2, T3, and T4, respectively. These results indicate that the threshold effects were not significant.

The AUCs of the SROC curve were 0.98 (95% CI = 0.97–0.99) for T1, 0.87 (95% CI = 0.84–0.90) for T2, 0.89 (95% CI = 0.86–0.92) for T3, and 0.96 (95% CI = 0.94–0.98) for T4. The SROC curve along with the summary point and the 95% confidence and prediction contours is shown in **Figure 3**.

### Clinical Utility of DCEUS

**Figure 4:**
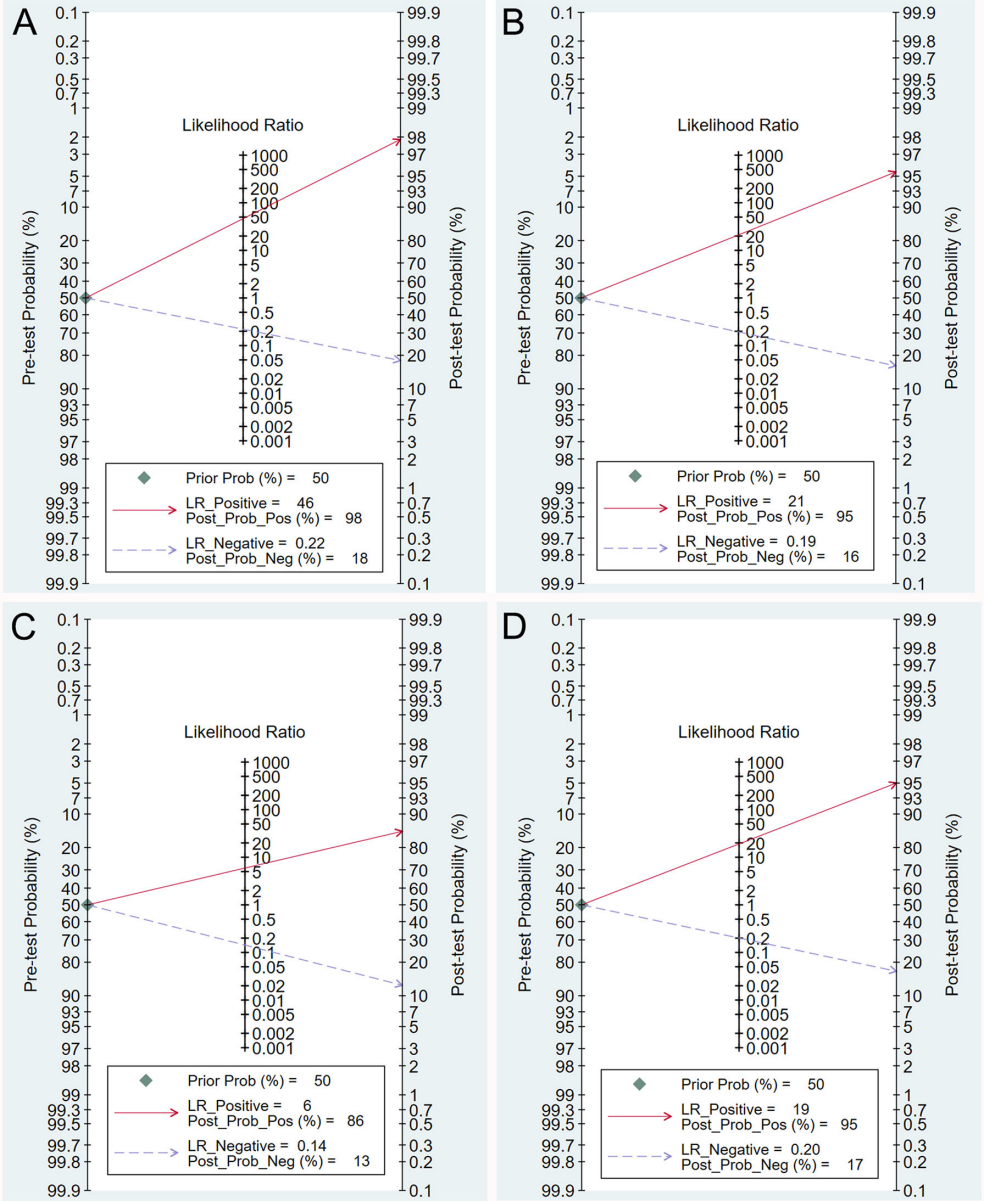
Fagan plot estimating how much the result of double contrast-enhanced ultrasonography changes the probability that a patient has a T1 **(A)**, T2 **(B)**, T3 **(C)**, or T4 **(D)** gastric cancer, considering a given pre-test probability (here the mean pre-test probability found in eligible studies is shown as an example).

The Fagan graph was plotted to show the relationship among the pretest probability, the likelihood ratio, and the posttest probability. When the pretest probability was set at 50%, the posttest probability was 98% if the results were positive and 18% if the results were negative for T1 tumors (**Figure 4A**). The posttest probability was 95% if the results were positive and 16% if the results were negative for T2 tumors (**Figure 4B**). The posttest probability was 86% if the results were positive and 13% if the results were negative for T3 tumors (**Figure 4C**). The posttest probability was 95% if the results were positive and 17% if the results were negative for T4 tumors (**Figure 4D**).

**Figure 5:**
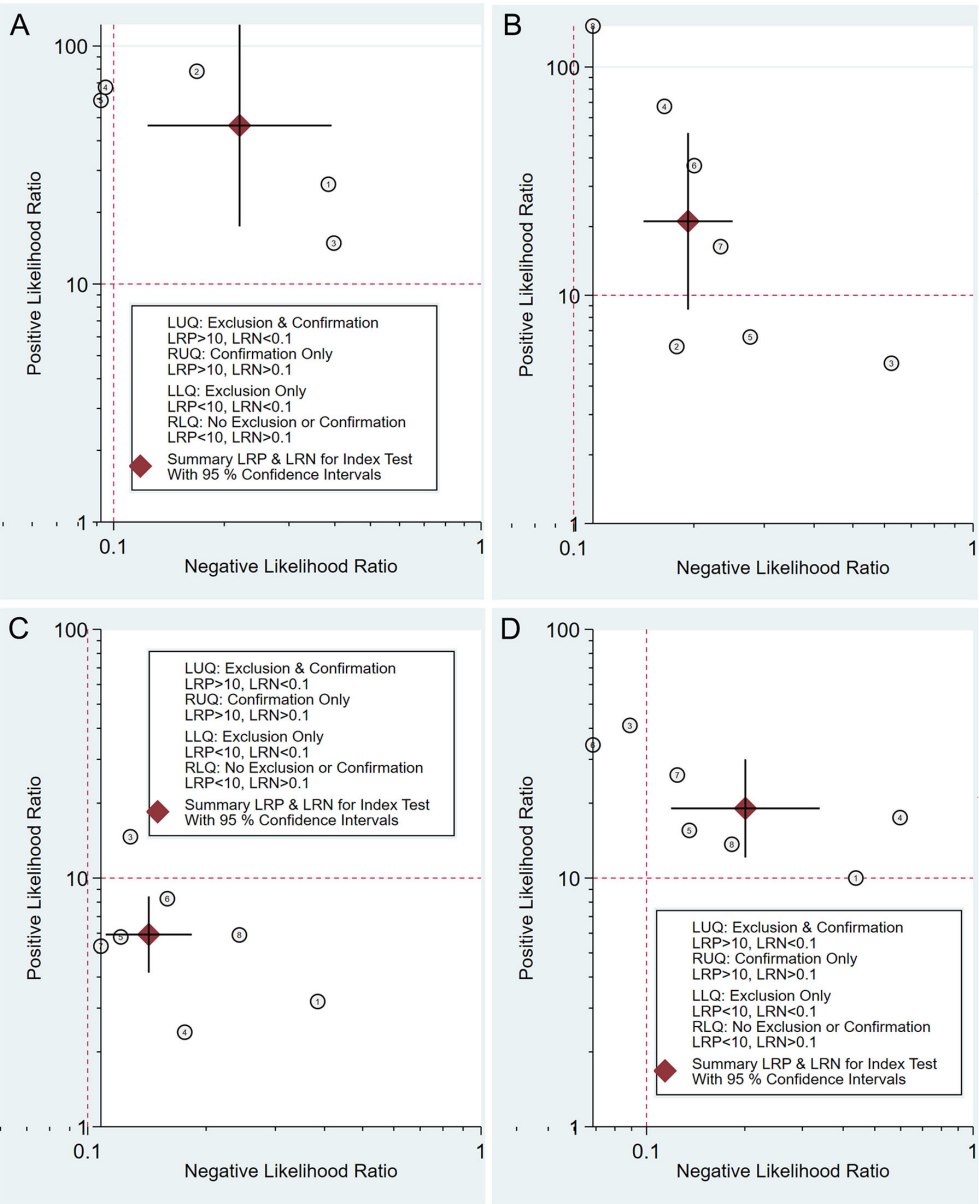
Double contrast-enhanced ultrasonography performance to diagnose T1 **(A)**, T2 **(B)**, T3 **(C)**, and T4 **(D)** gastric cancer. Likelihood ratio (LR) scattergram defining the quadrants of informativeness based on desirable thresholds (positive LR > 10, negative LR < 0.1): left upper quadrant (test suitable both for diagnosis exclusion and confirmation), right upper quadrant (confirmation only), left lower quadrant (exclusion only), and right lower quadrant (neither confirmation nor exclusion).

The likelihood ratio scatter plots demonstrated that the summary point of the probability ratio fell in the upper right quadrant for T1, T2, and T4, indicating that DCEUS was effective for T1, T2, and T4 confirmation but not effective for T1, T2, or T4 exclusion (**Figures 5A, B, D** **)**. In addition, the summary point of the probability ratio fell in the lower right quadrant for T3, indicating that the utility of DCEUS was limited for T3 evaluation (**Figure 5C**).

### Comparison of DCEUS *vs*. CT

**Figure 6:**
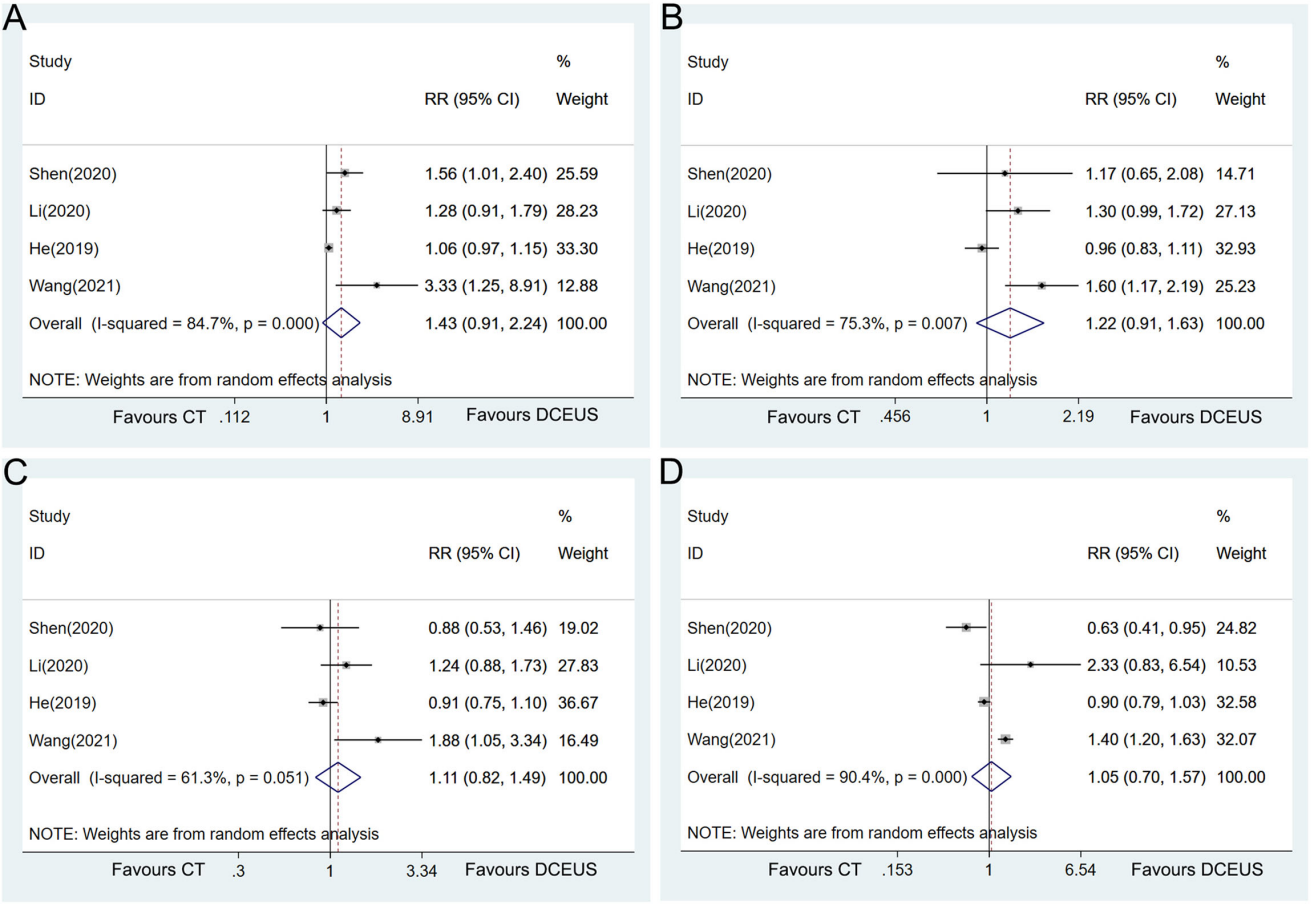
Forest plot showing the pooled effects of diagnostic performance of double contrast-enhanced ultrasonography compared with CT in diagnosing T1 **(A)**, T2 **(B)**, T3 **(C)**, and T4 **(D)** gastric cancer.

Data were available in 4 studies on the comparison of the diagnostic accuracy in determining the T stage of GC between DCEUS and CT. The pooled analysis failed to show a statistically significant difference between the two examinations in T1 (RR = 1.43, 95% CI = 0.91–2.24, *p* = 0.119, **Figure 6A**), T2 (RR = 1.22, 95% CI = 0.91–1.63, *p* = 0.177, **Figure 6B**), T3 (RR = 1.11, 95% CI = 0.82–1.49, *p* = 0.498, **Figure 6C**), or T4 (RR = 1.05, 95% CI = 0.70–1.57, *p* = 0.822, **Figure 6D**). Heterogeneity was detected in the pooled analyses in each T stage tumor (T1: *I* ^2^ = 84.7%, *p* < 0.001; T2: *I* ^2^ = 75.3%, *p* = 0.007; T3: *I* ^2^ = 61.3%, *p* = 0.051; and T4: *I* ^2^ = 90.4%, *p* < 0.001, **Figure 6**).

**Figure 7:**
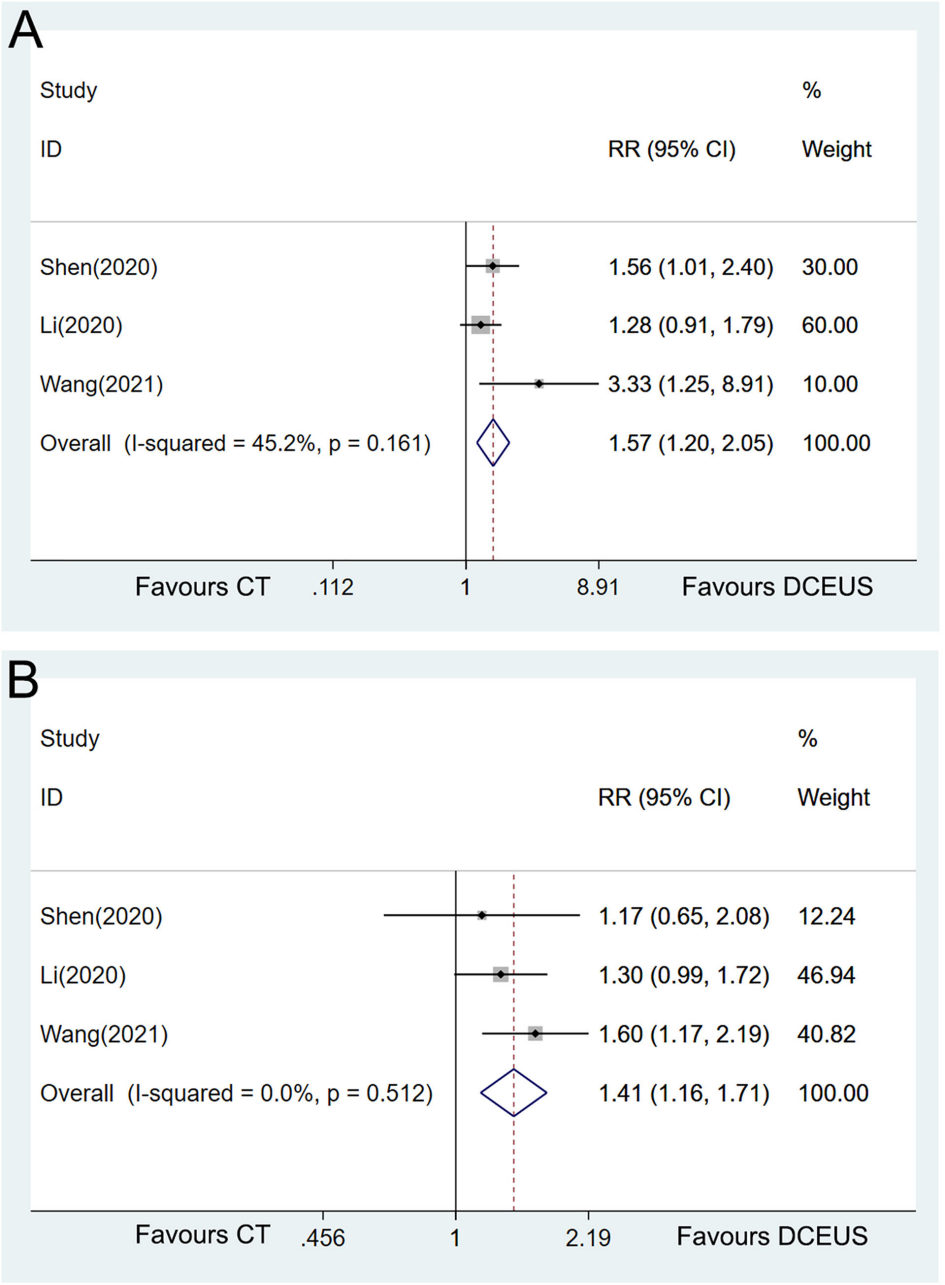
Forest plot showing the pooled effects of diagnostic performance of double contrast-enhanced ultrasonography compared with CT in diagnosing T1 **(A)** and T2 **(B)** with only homogeneous studies included.

Sensitivity analyses were implemented to explore the heterogeneity by omitting the included studies one by one. Interestingly, when the study by He et al. (13) was excluded from the pooled analyses, DCEUS demonstrated significant superiority to CT in diagnosing T1 (RR = 1.57, 95% CI = 1.20–2.05, *p* = 0.001, **Figure 7A**) and T2 (RR = 1.41, 95% CI = 1.16–1.71, *p* = 0.001, **Figure 7B**) with homogeneity.

### Comparison of DCEUS *vs*. EUS

**Figure 8:**
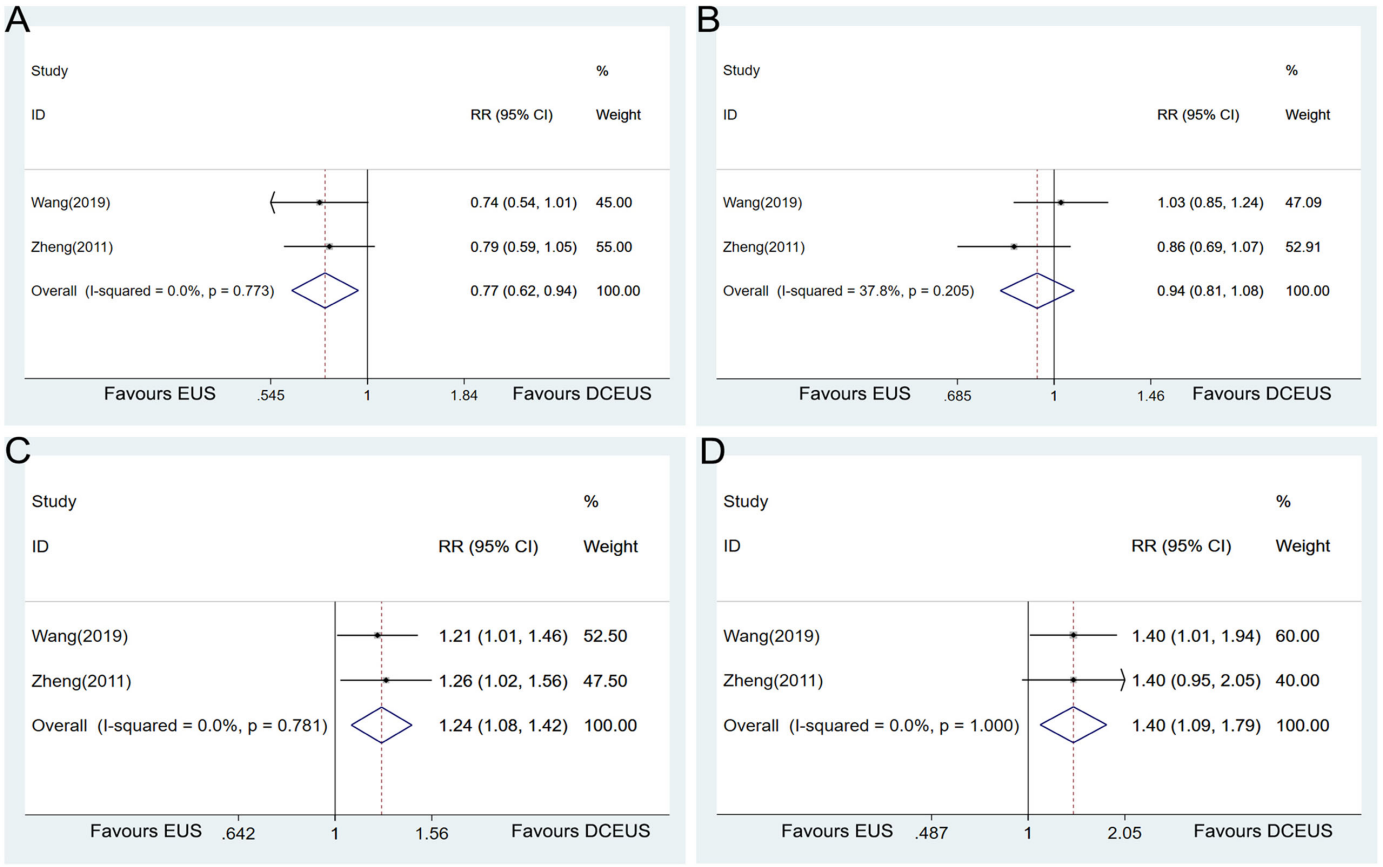
Forest plot showing the pooled effects of the diagnostic performance of double contrast-enhanced ultrasonography compared with endoscopic ultrasound in diagnosing T1 **(A)**, T2 **(B)**, T3 **(C)**, and T4 **(D)** gastric cancer.

Data were available in 2 studies on the comparison of the diagnostic accuracy in determining the T stage of GC between DCEUS and EUS. The pooled analysis showed that DCEUS had a lower diagnostic accuracy than EUS in T1 tumors (RR = 0.77, 95% CI = 0.62–0.94, *p* = 0.013, **Figure 8A**) but had a higher accuracy in T3 (RR = 1.24, 95% CI = 1.08–1.42, *p* = 0.003, **Figure 8C**) and T4 tumors (RR = 1.40, 95% CI = 1.09–1.79, *p* = 0.008, **Figure 8D**). No significant difference was detected in the T2 tumors between the two examinations (RR = 0.94, 95% CI = 0.81–1.08, *p* = 0.370, **Figure 8B**).

### Publication Bias

Deeks’ test was applied to assess the publication bias. The *p*-value was 0.015, 0.325, 0.111, and 0.987 for T1, T2, T3, and T4, respectively, indicating the presence of publication bias.

## Discussion

Precise preoperative staging is greatly essential for proper stage-dependent patient management (27). It is utilized to select patients who may benefit from less invasive diagnostic procedures and those who may benefit from multimodal treatment (28). This systematic review provides an overview of current evidence on the diagnostic performance of DCEUS for preoperative T staging in patients with GC. On the whole, the sensitivity, specificity, and AUC of DCEUS in the diagnosis of each T stage of GC are relatively high. This information enables clinicians to get a precise sense of the risk of making errors, in terms of both false-positive and false-negative predictions. More importantly, DCEUS shows a superiority to CT in the diagnosis of stages T1 and T2 tumors and to EUS in stages T3 and T4 tumors. Therefore, DCEUS could serve as a feasible complementary diagnostic tool for the clinical T staging of GC.

Currently, MDCT is the most commonly used imaging method for staging GC, which can provide visualization of the depth of the primary tumor invasion and an estimate of the lymph node involvement (29). However, the diagnostic performance of CT for T staging is limited, especially for early GC (30). DCEUS was developed as a novel method to complement existing modalities in the staging of GC. It is based on oral gastric window contrast-enhanced ultrasonography and further uses ultrasound venography to analyze the blood flow perfusion of the lesion. The diagnostic method zywas based on the characteristics of “positive imaging” in the arterial phase and “negative imaging” in the venous phase and T staging were performed according to the range of these two areas (31). In the present meta-analysis, DCEUS reveals superiority to CT in the diagnosis of T1 and T2 stage of GC, and such difference reaches statistical significance in the study by He et al. (13), which contributes to the heterogeneity, and is excluded from the pooled analysis. These results were consistent with the previous meta-analysis by Xu et al. (16). Since only a small number of patients are included, these inspiring results would trigger more clinical studies to further elucidate the diagnostic performance of DCEUS.

EUS is routinely used in the preoperative staging of GC since remarkably different echogenic appearances could be displayed between the adjacent structural layers of the stomach (32). All available guidelines on GC recommend EUS as the main method to assess the T stage (33). The diagnostic accuracy of EUS for overall T staging varied from 56.9 to 87.7%, and the accuracy values for T1, T2, T3, and T4 stages were 14 to 100%, 24 to 90%, 50 to 100%, and 25 to 100%, respectively (34–37). In the present meta-analysis, two original studies (15, 25) reported the head-to-head comparison results between DCEUS and EUS in the diagnosis of T staging of GC. DCEUS yields a better consistency with postoperative pathological results than EUS in T3 and T4 tumors, and EUS seems to have a higher accuracy in diagnosing T1 tumors than DCEUS. Despite the inspiring results, DCEUS should only be considered as a research method, an alternative tool, and may not be used as a routine procedure for GC staging.

There was a meta-analysis by Xu et al. published previously on the diagnostic accuracy of DCEUS in clarifying the tumor depth of GC (16). In that publication, a total of 926 patients from 6 studies were included, and the pooled sensitivity and specificity of DCEUS were 0.67 and 0.98 for T1 stage, 0.81 and 0.95 for T2 stage, 0.89 and 0.86 for T3 stage, and 0.87 and 0.96 for T4 stage, respectively. However, some defects exist in that meta-analysis. Data from the studies by Chen et al. (26) and Wang et al. (38) were based on the overlapping population, and three most recently published relevant studies were not included, which makes the pooled results less convincing. More importantly, they did not provide the pooled comparison of the diagnostic performance between DCEUS and other tools (CT or EUS). Therefore, our present meta-analysis may serve as an updated and revised version.

Despite these favorable findings, some critical issues need to be emphasized to correctly appreciate the limitations of DCEUS. Firstly, the remarkable heterogeneity of results across eligible studies casts some doubts on the reliability and reproducibility of DCEUS in the tumor staging of GC. Since the study by He et al. contributed a great amount to the heterogeneity, it seems that the ultrasonography equipment utilized and the differences in the experience levels of the doctors performing the ultrasound examination might be the factors that brought about the heterogeneity. However, we could not explore the effect of other potential sources of heterogeneity due to the lack of data. Secondly, all the included studies were conducted among Chinese populations, and no data derived from Caucasians or black people are available. As the thickness of abdominal fat in Chinese patients is thinner than those in people from Western countries, it may be beneficial to obtain clear images when performing the DCEUS examination. Therefore, the generalizability of the findings to a population with different races, ethnicity, or geographical environments may be limited. Finally, since only a small number of studies and patients are available to make a pooled analysis, these findings should be interpreted with caution. More restrictedly designed studies are still warranted to make a direct comparison of DCEUS with CT or EUS to further confirm the clinical utility value of DCEUS.

## Conclusions

The findings obtained from the present meta-analysis provide evidence for the utility of DCEUS in the preoperative tumor staging of GC. DCEUS showed a superiority to CT in the diagnosis of stage of T1 and T2 tumors and to EUS in the staging of T3 and T4 tumors. Therefore, DCEUS could serve as a feasible complementary diagnostic tool for clinical T staging of GC. However, it is still far from a definitive conclusion for DCEUS to be proposed for use in routine clinical practice.

## Data Availability Statement

The original contributions presented in the study are included in the article/**Supplementary Materials**. Further inquiries can be directed to the corresponding authors.

## Author Contributions

WW and HH designed this study. XZ, JY, and YZ acquired and analyzed the data. XZ and XH wrote and edited the manuscript. All authors contributed to the article and approved the submitted version.

## Funding

This study was supported by grants from the National Natural Science Foundation of China (81773049, 81402359, and 81602617).

## Conflict of Interest

The authors declare that the research was conducted in the absence of any commercial or financial relationships that could be construed as a potential conflict of interest.

## Publisher’s Note

All claims expressed in this article are solely those of the authors and do not necessarily represent those of their affiliated organizations, or those of the publisher, the editors and the reviewers. Any product that may be evaluated in this article, or claim that may be made by its manufacturer, is not guaranteed or endorsed by the publisher.

## Supplementary Material

Supplementary Figure S1 Forest plot to show the pooled effects of diagnostic performance of DCEUS in diagnosing stage T1 gastric cancer.

Supplementary Figure S2 Forest plot to show the pooled effects of diagnostic performance of DCEUS in diagnosing stage T2 gastric cancer.

Supplementary Figure S3 Forest plot to show the pooled effects of diagnostic performance of DCEUS in diagnosing stage T3 gastric cancer.

Supplementary Figure S4 Forest plot to show the pooled effects of diagnostic performance of DCEUS in diagnosing stage T4 gastric cancer.

Supplementary Figure S5 Results of the sensitivity analysis of the pooled effects of diagnostic performance of DCEUS in diagnosing stage T1 **(A)**, T2 **(B)**, T3 **(C)**, and T4 **(D)** gastric cancer by removing each study one by one.
