# Supplementary figures and images for: Updated Evaluation of the Diagnostic Performance of Double Contrast-Enhanced Ultrasonography in the Preoperative T Staging of Gastric Cancer: A Meta-Analysis and Systematic Review
Source: Front Oncol. 2022 Mar 9;12:844390. doi: 10.3389/fonc.2022.844390 (PMC8959463; doi:10.3389/fonc.2022.844390)

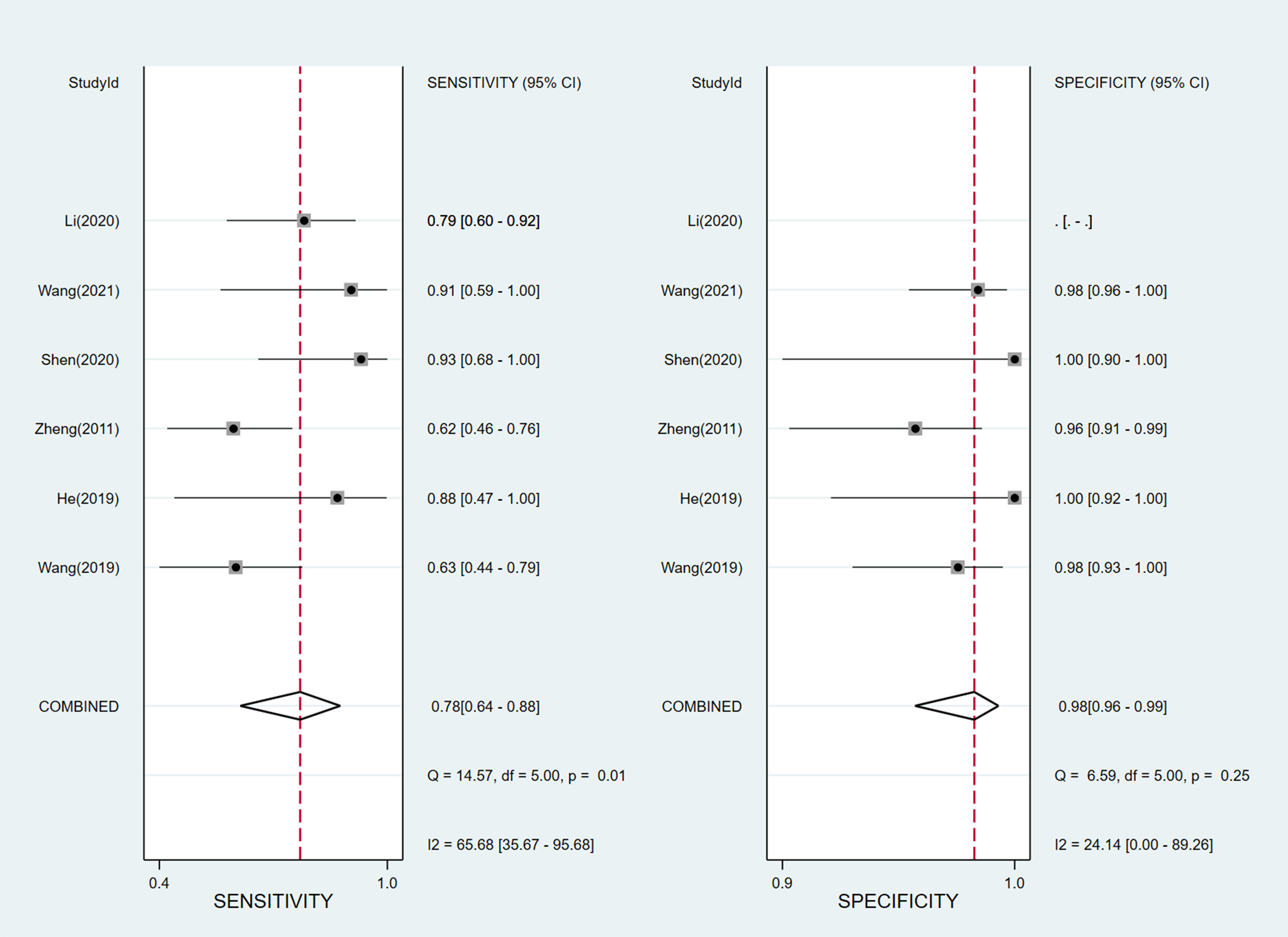

Supplement: Supplementary Figure S1 — Forest plot to show the pooled effects of diagnostic performance of DCEUS in diagnosing stage T1 gastric cancer. [file Image_1.jpeg]

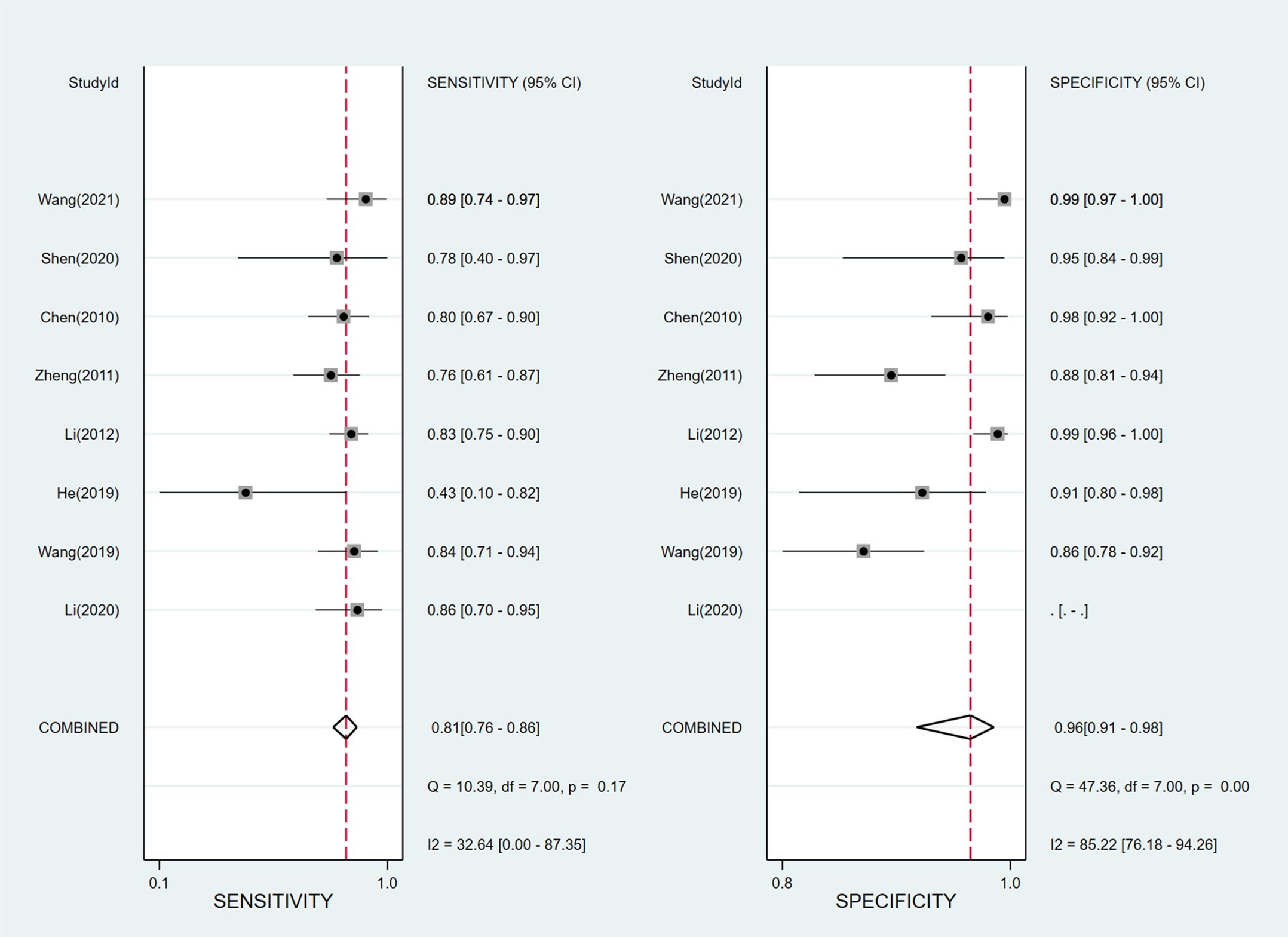

Supplement: Supplementary Figure S2 — Forest plot to show the pooled effects of diagnostic performance of DCEUS in diagnosing stage T2 gastric cancer. [file Image_2.jpeg]

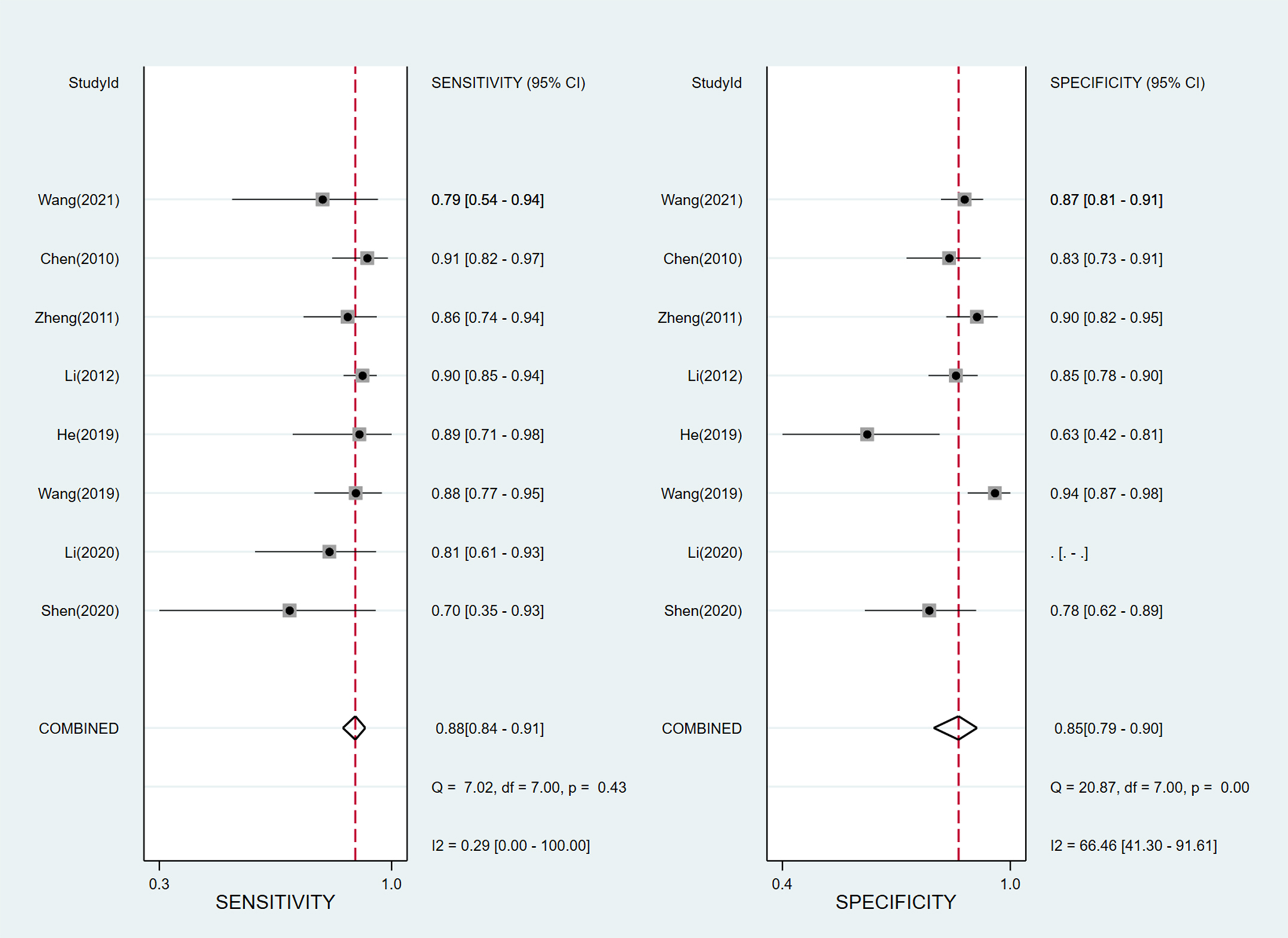

Supplement: Supplementary Figure S3 — Forest plot to show the pooled effects of diagnostic performance of DCEUS in diagnosing stage T3 gastric cancer. [file Image_3.jpeg]

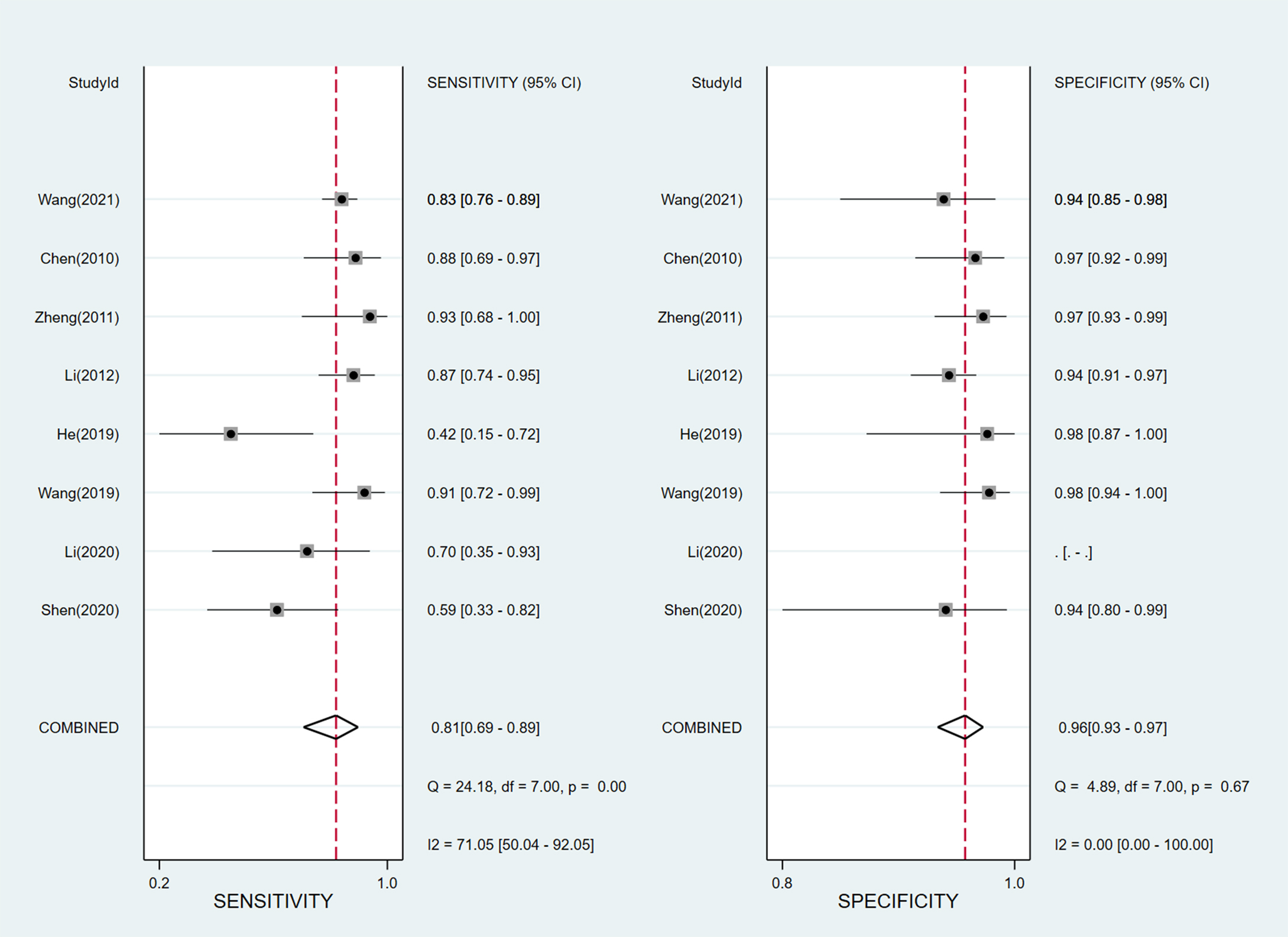

Supplement: Supplementary Figure S4 — Forest plot to show the pooled effects of diagnostic performance of DCEUS in diagnosing stage T4 gastric cancer. [file Image_4.jpeg]

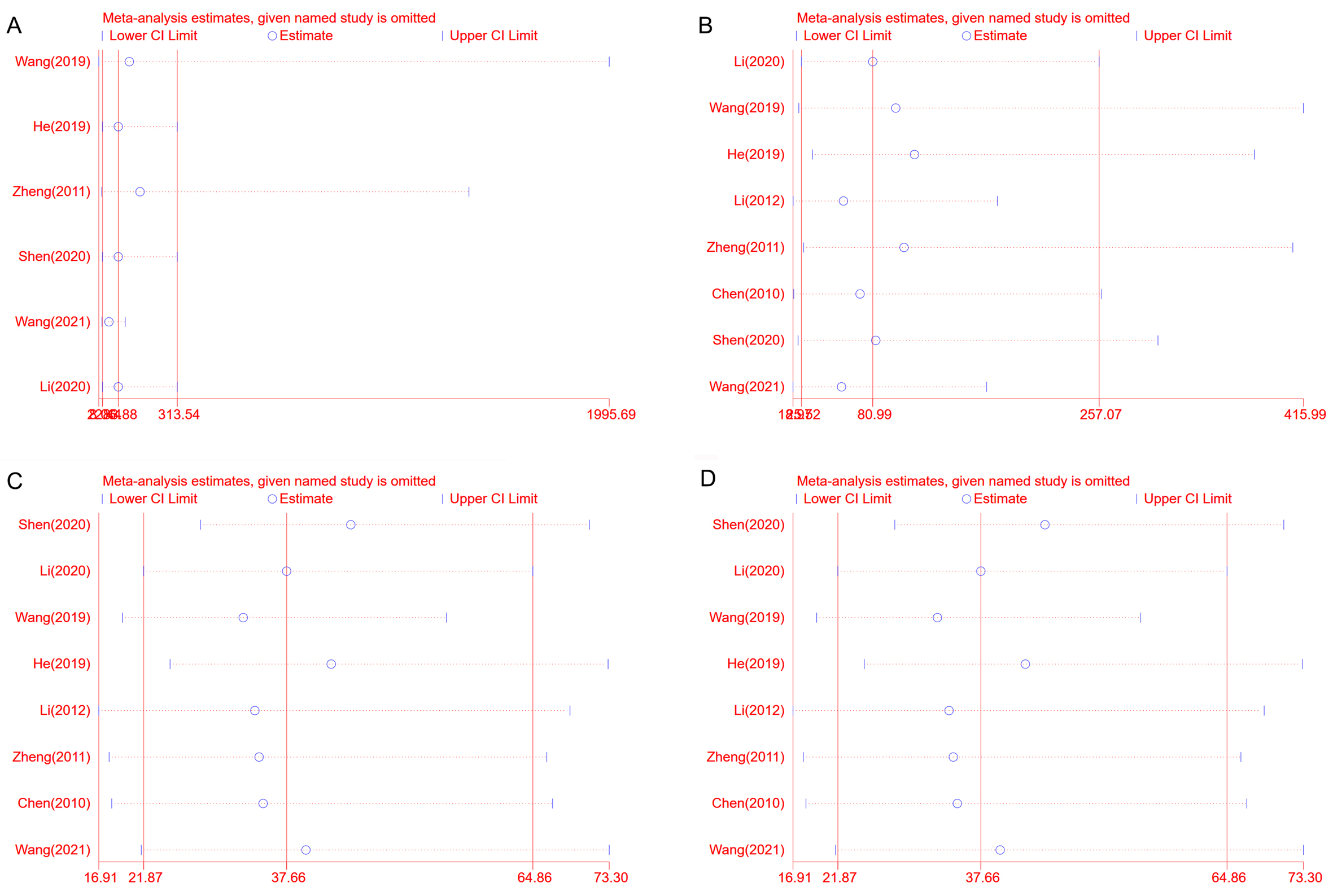

Supplement: Supplementary Figure S5 — Results of the sensitivity analysis of the pooled effects of diagnostic performance of DCEUS in diagnosing stage T1 (A), T2 (B), T3 (C), and T4 (D) gastric cancer by removing each study one by one. [file Image_5.jpeg]
